# Perceptions of Justice By Algorithms

**DOI:** 10.1007/s10506-022-09312-z

**Published:** 2022-04-05

**Authors:** Gizem Yalcin, Erlis Themeli, Evert Stamhuis, Stefan Philipsen, Stefano Puntoni

**Affiliations:** 1 Rotterdam School of Management, Erasmus University Rotterdam, Postbus 1738, 3000 DR Rotterdam, Netherlands; 2 Erasmus School of Law, Erasmus University Rotterdam, Postbus 1738, 3000 DR, Rotterdam, Netherlands; 3 Montaigne Centre for Rule of Law and Administration of Justice, Utrecht University, Heidelberglaan 8, 3584 CS Utrecht, Netherlands

**Keywords:** Justice, Algorithms, Trust, Complexity, Civil court, Automation

## Abstract

Artificial Intelligence and algorithms are increasingly able to replace human workers in cognitively sophisticated tasks, including ones related to justice. Many governments and international organizations are discussing policies related to the application of algorithmic judges in courts. In this paper, we investigate the public perceptions of algorithmic judges. Across two experiments (N = 1,822), and an internal meta-analysis (N = 3,039), our results show that even though court users acknowledge several advantages of algorithms (i.e., cost and speed), they trust human judges more and have greater intentions to go to the court when a human (vs. an algorithmic) judge adjudicates. Additionally, we demonstrate that the extent that individuals trust algorithmic and human judges depends on the nature of the case: trust for algorithmic judges is especially low when legal cases involve emotional complexities (vs. technically complex or uncomplicated cases).

**Supplementary Information:**

The online version contains supplementary material available at 10.1007/s10506-022-09312-z.

## Introduction

Since the 1960s, scholars have been discussing the use of computers for analysing and predicting judicial decisions (Elardo 1968; Lawlor 1963). They proposed that computer programs can not only find and analyse the law but also can predict decisions (Lawlor 1963). Even though computers have not yet reached widespread adoption in courts in the way these scholars envisioned, advancements in technology have recently started to enable the automated processing of large quantities of data as well as the handling of complex tasks (Parmar et al. 2014).

Artificial intelligence (AI) technology is rapidly spreading in our society (Granulo et al. 2019; Ostrom et al. 2015; Rust and Huang 2014). AI can provide personalized advice (Logg et al. 2019; Yeomans et al. 2019), interact with customers (Van Doorn et al. 2017), and drive vehicles autonomously (Lafrance 2015). In addition to their adoption in everyday life and businesses, AI has been increasingly used in government services (Feldstein 2019; Liu et al. 2019; Sun and Medaglia 2019; Mehr 2017). Today, computational, and predictive technologies are already being used in medicine (Longoni et al. 2019), education (Tuomi et al. 2018), military (Cummings 2017), and justice systems (Branting et al. 2021; Fry 2018).

With the increasing use of algorithms and AI by law firms, courts have become more familiar with this technology. Law firms use algorithms and AI to read documents, prepare case files, and predict the win rate of court cases (Faggella 2020; Donahue 2018). It is safe to expect that the current state is just the beginning stage of AI application in courts. Recent reports indicate that AI can already forecast court decisions with great accuracy (Quattrocolo 2020; Katz et al. 2017; Sulea et al. 2017; Aletras et al. 2016). In particular, algorithms have been shown to handle simple and standard cases (Mandri 2019), which consists of the vast majority of legal case load (Quattrocolo 2020; Commission for the Evaluation of the Efficiency of Justice 2019; Pagallo and Durante 2016; Silvestri 2014; Uzelac 2014; Reiling 2010). Unsurprisingly, new systems (e.g., Branding et al. 2021; Ruggeri et al. 2021; Bagherian-Marandi et al. 2021) are being developed that aim directly at resolving conflicts by AI. Limiting ourselves to the North Atlantic, an increasing number of governments (e.g., Estonia, England, the Netherlands) and international organizations (e.g., the Council of Europe) have been discussing and formulating policies related to the application of algorithmic decision-makers in courts (Castelluccia and Le Métayer 2019; Mandri 2019; Dekker 2018). In particular the European Union (EU), has acknowledged the benefits of using AI in the justice sector (European Union 2018), but it has also highlighted the dangers of deploying AI in the justice sector may be difficult to anticipate, identify or measure (High-Level Expert Group on Artificial Intelligence 2019). To mitigate these dangers and maximise the benefits of AI, trust in algorithmic systems is of outmost importance (European Commission 2019). The result of these discussions was the creation of an Assessment List for Trustworthy Artificial Intelligence (ALTAI) for self-assessment by the High-Level Expert Group on Artificial Intelligence of EU. Following these steps and in view of the importance of courts, the European Commission’s proposal for an AI regulation categorises AI used in court as high-risk. High-risk AI systems will have special requirements for data governance, document and record keeping, transparency requirements, and human oversight. It is difficult to speculate on the impact of this proposed regulation on the deployment of AI in court but increasing trust in AI systems will certainly benefit their adoption.

When the algorithmic judges are applied, many important legal questions will be raised. How will adoption of algorithms and AI influence the role of human judges? How will the adoption of algorithmic judges impact citizens’ trust in the court system? How will adoption of algorithms that resolve disputes influence individuals’ willingness to submit their legal cases to a court? What are the possible advantages and disadvantages of such algorithmic judges in the public’s eye? We argue that any potential future decision on the adoption or the development of such algorithmic judges should take the perceptions and intentions of potential court users into account. Small and uncontested claims are most likely type to be resolved by algorithmic judges. Parties in small and uncontested claims are often self-represented and do not use any form of legal advice. Thus, this type of user is often marginalised and overlooked when drafting legal reforms or adopting new technologies. To the best of our knowledge, there is no scientific research available on court users’ perception of technological applications taking a decisive share in the adjudication. However, one study (Helberger et al. 2020; Araujo et al. 2018) investigates individuals’ reaction towards automated decision makers in the justice field. While the studies share many elements, there are also many differences which we elaborate in the discussion.

In current work, we study individuals’ perceptions towards algorithms deployed in judicial decision-making. As the public’s trust in the justice administration is an important benchmark for a good government (Karpen 2010) and is often used as a reference point for the quality of the protection of the rule of law, we therefore investigate how interacting with algorithmic (vs. human) judges affects the extent that individuals trust them. We also test whether there are downstream consequences of changes in trust, such as individuals’ intentions to submit their legal cases to the local court. Additionally, we test whether perceptions of trust are affected by the complexity of a legal case. Finally, we investigate individuals’ awareness of potential advantages of algorithmic judges (i.e., speed, cost) over human judges.

Legal literature is rich in studies about the crisis of civil justice and the lack of access to justice (Cappelletti et al. 1978; Biard et al. 2021). Civil courts are frequently congested and have trouble with the increasing number of cases and their growing complexity. While court users face higher costs and more complex procedures. The crisis was exacerbated by the 2008 financial crisis which obliged governments to cut costs and develop strategies to deal with the ever present access to justice issue. As a result, governments are pushing towards specialised courts which should deal with complex high value commercial cases, consider for example the rise of international commercial courts and the specialised chambers around Europe (Kramer and Sorabji 2019). Ideally, these specialised courts should deal faster and more efficiently with complex cases and, often, they will have higher fees to be financially independent (Aran and Ofir 2020). On the other end, low value non-complex cases are being pushed outside of the courts voluntarily because courts do not have the capacity to deal with them and involuntarily because barriers to access courts are increasing. Obviously, this is problematic because people cannot find redress for their problems, and those that use alternative dispute solutions or online dispute resolutions do not have any guarantee about their quality and standards. It is not surprising that court administrators consider AI as a potential solution to this situation (Schmitz 2019; Kramer 2016). Our research adds to the growing number of studies on the use and effects of AI in courts (Micklitz et al. 2021; Forrest 2021; de Souza and Spohr 2021). In particular, our study brings empirical evidence on the reaction of court users when facing an algorithmic judge. This is very valuable for both court administrators and software developers. Court administrators can use the data from our study to better deploy such software, make more effort to increase trust, better advertise court digitisation, conduct follow-up studies with their constituents. Software developers can use our study to see what are the elements that court users consider more problematic or more beneficial, in what type of cases, but also run their own studies to better determine how to develop their products.

## Trust in Judges

Existing work reveals the public’s trust in courts as an essential component of good governance (Jackson et al. 2011; Karpen 2010; Savela 2006), and citizens’ trust in government organizations impacts their intentions (e.g., willingness to report a crime; Bennett and Wiegand 1994; Silver and Miller 2004). Given such importance of perceived trust (Canal et al. 2020; Elliot and Arthur 2020), many governments and international institutions monitor and try to improve public trust (The Danish Court Administration 2015). Trust in institutions becomes even more important at a time when reports show that public trust has been declining due to economic distress, agitation and propaganda spread through social media, and demagogy politics (Hutchens 2018).

Previous work documents a strong connection between court users’ evaluation of how they are treated and their trust in judges (Tyler et al. 2019; Grootelaar and Van den Bos 2018; Lind 2018; Van den Bos et al. 2014; Lind et al. 1993). In line with the previous literature, we propose that perceived trust towards (algorithmic or human) judges is an important factor that policy-makers and governments should consider. Next, we identify essential factors of perceptions of trust in the existing research.

To earn and maintain public trust, courts should foremost fulfil their functions (Resnik 2013; Genn 2009; Mnookin and Kornhauser 1979). Two of the most important factors that affects citizens’ trust in courts and the legal system is the extent that judicial officers are fair and unbiased (Rottman and Tyler 2014; Warren 2000). Judges are expected to perform all their duties in an unbiased and fair way, and to treat everyone equally (Martyn et al. 2017; Rädler 1997). Previous research also states that fairness and unbiasedness are strongly correlated and greatly impact perceptions of justice (Nagtegaal 2021; Helberger et al. 2020; Elliot and Arthur 2020; Lind at al. 1990). It is no surprise then that recent reports list perceived fairness and impartiality to be influential in affecting the choice of court (IPSOS 2019; BlackBox Research Pte 2016; Lein at al. 2015).

In addition to these two factors, legal stability and predictability are also fundamental to what people mean by “the rule of law” (Resnik 2013; Genn 2009; Schwarzschild 2007; Mnookin and Kornhauser 1979). Predictability, for instance, has a moral valence as it assures that cases will be treated equally based on an existing law (Lindquist and Cross 2012). When judges act unpredictably, it does not only damage individuals’ trust in the legal system, but also creates a less stable legal environment for the development of economic and other human relations (Lindquist and Cross 2012).

Reviewing the literature on trust and procedural justice, we consider perceived trust as a combination of court user’s perception of predictability, fairness, trustworthiness, and unbiasedness of a judicial decision-maker. Supporting the relevance of these dimensions, a recent survey found that fairness and predictability of the outcome, impartiality are found to be among the factors that influences the decision of going to a court the most (Themeli 2018).

## Algorithmic versus Human Judges

Existing work has documented several systematic differences in individuals’ perceptions of algorithmic versus human decision-making (Helberger et al. 2020; Yeomans et al. 2019). Looking at this stream of research, there are both upsides and downsides of algorithmic decision-makers. For instance, algorithms might be perceived as more consistent and objective than humans (Helberger et al. 2020; Lee 2018); however, individuals also think that algorithms (vs. humans) tend to ignore their unique characteristics (Longoni et al. 2019) and are less authentic (Jago 2019).

Considering individuals’ trust towards algorithmic and human decision-makers, general finding in this line of research is that even though algorithms objectively outperform humans (Kaufmann and Wittmann 2016; Grove et al. 2000; Camerer 1981; Meehl 1954), individuals are often reluctant to rely on algorithms (Yeomans et al. 2019; Dietvorst et al. 2015; Dzindolet et al. 2003; Dawes et al. 1989). For instance, individuals trust a human advisor (e.g., doctor) more than an algorithmic advisor (Longoni et al. 2019; Promberger and Baron 2006). In the field of online dispute resolution (ODR), recent work by Sela (2018) documents individuals’ positive perception of procedural justice when an online software is used as mediator, but a negative perception of procedural justice when an online software is used as arbitrator (Sela 2018). Regarding possible reasons for such aversion, previous work suggests that algorithm aversion could stem from a variety of reasons including individuals’ desire for control over outcomes (Dietvorst et al. 2018), the perception that humans are easier to understand (Yeomans et al. 2019), or the opportunity to provide input into the dispute resolution and the consistency of treatment (Sela 2018). Conversely, Helberger et al. (2020) find that people may consider automated decision-makers more fair than human decision-makers. This study suggests that emotions, the risk of manipulation, the need for a human touch, and the need to consider the context were important elements that influence how fair humans or automated decision-makers were considered. However, Helberger et al. (2020) indicate that other variables may play a role when comparing a human and automated decision-makers, which indicates the complexity of human algorithm interaction. In addition to this, Rule and Friedberg argue that trust in a particular ODR system is not build solely on the merits of that dispute resolution system but borrows from the environment where the dispute resolution system operates (Rule and Friedberg 2005). In line with these findings, we hypothesize that individuals will trust algorithmic judges less compared to human judges in a divorce case and a small civil claim. We assume that the judicial system is stable and there is no visible dissatisfaction from it. Additionally, we expect this lack of trust to have downstream consequences and lead to lower intentions to submit cases to a court.

Despite these predictions, there might still be perceived benefits in using algorithms as judges. We expect individuals to acknowledge some of the advantages of algorithmic judges. For instance, algorithms can be expected to complete the same task faster than humans due to their optimized procedures and high processing capabilities (Soltanian-Zadeh et al. 2019; Schneider et al. 2018). Moreover, adoption of technologies often leads to drastic reduction of operation cost as algorithms and machines do not require compensation (e.g., salary, pension fund, insurance; Meuter et al. 2000). Accordingly, we expect individuals to acknowledge these advantages and perceive algorithmic judges to be cheaper and faster than human judges.

## Case Complexity

Previous research on algorithmic decision-making, suggests that the type of task at hand impacts individuals’ attitudes towards the decision-maker (algorithms versus humans; Castelo et al. 2019). One classification that is often used is whether a task is emotional or cognitive in nature (Castelo et al. 2019; Waytz and Norton 2014). This stream of research indicates that non-human entities (e.g., organizations, robots) are perceived to be capable of thinking, but not feeling (Rai and Diermeier 2015; Gray and Wegner 2012). Accordingly, individuals are shown to express more favourable attitudes towards algorithms when a task is framed as requiring cognition compared to emotion (Waytz and Norton 2014; Lee 2018). Building on this work, we test whether individuals trust algorithmic and human judges differently depending on the nature of a legal case. Specifically, we propose that court users may perceive algorithmic judges especially more negatively (i.e., low perceived trust) when the legal case contains complexities that arise from psychological and emotional factors, compared to cases low in complexity or cases where complexity arises from technical issues.

The concept of case complexity has taken on increased theoretical importance over the years (De Vey Mestdagh 2020; Campbell and Gingrich 1986; Campbell 1984). According to the existing work in this literature, complexity can come from many different sources (De Vey Mestdagh 2020; Campbell 1988; Earley 1985; Huber 1985): for instance, complexity can originate from psychological factors (e.g., identity relevance) or technical factors (e.g., number of rules to follow). We do not intend to discuss the definition of complexity here, but we would like to highlight the factors that increase complexity. As we detail in the present paper, our experiment starts with a case where parties agree about its outcome and do not have any conflict between them. We consider this a simple case and the type of case where an AI-judge can best be deployed (Pagallo and Durante 2016). With the intention to make it more complex, we add to this simple case some legal elements that do not change its outcome. We do the same by adding an emotional element to the simple case to make it more emotionally complex. This way, when we mention case complexity, we refer to the added elements that the simple case received.

## Overview of Experiments

Across two experiments (N = 1,822), we examine how algorithmic (vs. human) judges affect trust. In our studies, participants read the description of a situation and are asked to complete a survey about their reactions. The materials were designed for a general audience and were written in non-technical language. We also used a fictional legal situation that is common in courts: a divorce case. We provided participants with the background of a legal case and randomly assigned them to either a human or an algorithmic judge. We also manipulated the type of case complexity (low vs. high emotional vs. high technical complexity): Participants in the low complexity condition were given a straightforward and simple case description, whereas we added details to complicate the case in the remaining conditions. Specifically, we either added technical (e.g., unequal shares of property) or emotional (e.g., psychological problems) details.

In each experiment, we measured participants’ trust towards their assigned judge by aggregating four items (i.e., perceived trustworthiness, unbiasedness, fairness, and predictability). We also measured participants’ willingness to submit the case to a local court. Finally, we measured perceived cost and speed of the judge. For the full list of measures and the scenario that were used in our experiments, see our Supplemental Materials. All the data and study materials are available at https://osf.io/z745a/?view_only=fda9280ab4354c10a5283e418ff7400c.

All participants were recruited using Mechanical Turk (Mturk), an online labour market operated by Amazon, the largest digital retailer. In the past few years, Mturk has become a leading source of human respondents for the behavioural sciences. Mturk has been shown to be a source of good data and has the advantage of enabling larger and more representative samples than many of the commonly used alternatives (e.g., student pools) (Paolacci and Chandler 2014). To make sure that participants were paying attention to the experimental stimuli and to ensure quality data, we included an attention check in the experiment. Only participants who answered the attention check correctly were directed to the study.

## Experiment 1

The main objective of experiment 1 is to test our main hypotheses that individuals trust algorithmic judges less than human judges and have lower intentions to submit their cases to the court. Additionally, this empirical study aims to test whether this perceived trust depends on the type of complexity (low complexity vs. high technical complexity vs. high emotional complexity) of the legal case.

### Design and Participants

We recruited 608 American Mturkers (M_age_ = 38.17, 50.8% F). Experiment 1 employed a 2 (judge type: algorithm vs. human) x 3 (case complexity type: low complexity vs. high technical complexity vs. high emotional complexity) between-participants design. Participants were randomly assigned to one of the six experimental conditions.

### Materials and Procedure

Participants were asked to imagine that they have been married for some years. As the love in their marriage has cooled down to almost zero, they and their partner agreed to separate and file for divorce. First, we manipulated the complexity of the divorce case. In the low complexity condition, participants were given an uncomplicated description of the divorce case (e.g., equal share of cost and property). In the high technical complexity condition, they were given a more complicated description in which the complexity arose from technical details (e.g., unequal shares of property, mortgage, inheritance). Finally, in the high emotional complexity condition, the complexity was due to emotional details (e.g., mental health problems of their partner). They were then given information about the judge that would take their divorce case. In the human judge condition, they were informed that cases like theirs are resolved by an experienced judge from the local court, whereas participants in the algorithmic judge condition were told of a new system, in place for some time, where cases like theirs are resolved by fully automated artificial intelligence and algorithms, that use the legislation and the relevant case law of their jurisdiction to resolve disputes.

After reading the scenario, participants indicated their general trust towards the judge. As reviewed in the literature, we compiled a scale of four items to capture perceived trust (1 = *unfair / biased / not trustworthy / unpredictable* to 9 = *fair / unbiased / trustworthy / predictable*; α = 0.84). We also measured how likely individuals were to submit their case to the local court (i.e., “How likely would you be to submit your case that will be resolved by the artificial intelligence (vs. judge) to the local court?”; 1 = *not at all likely* to 11 = *very likely*). Participants then filled out our manipulation check on perceived complexity of the legal case on a 11-point scale (i.e., “When you think about the case that you read, how complicated do you think this divorce case is?”, “How complicated do you think this divorce case is for artificial intelligence (vs. judge) to resolve?”; α = 0.76; 1 = *not at all complicated* to 11 = *very complicated*). Additionally, considering that speed and cost of a judge are factors that can influence attractiveness of a court (IPSOS 2019; Themeli 2018), we also measured perceived speed and cost of the judge (i.e., “Thinking about this divorce case and your future court experience, to what extent do you think that the artificial intelligence (vs. judge) will be ____; 1 = *slow/expensive* to 9 = *fast/cheap*). For ease of interpretation, in the analyses and graphs for all experiments below we reverse-coded the perceived cost item (1 = *cheap* to 9 = *expensive*), such that higher scores indicate higher perceived cost.

## Results of Experiments 1

Based on the measures discussed above, we computed indices by averaging the items used to measure each construct. These indices were then submitted to a General Linear Model where the two experimental factors and their interaction were entered as predictors.

### Manipulation Check

The main effect of case complexity was found to be statistically significant (*F*(2, 602) = 28.92, *p* < .001, η_p_^2^ = 0.09), and the contrast between high emotional complexity and high technical complexity cases were non-significant (*p* = .96), meaning that the perceived complexity of the complex cases was the same, regardless of its cause (i.e., technicality, psychological factors). Importantly, this contrast analysis also revealed that both high complexity conditions were perceived to be more complex than the low complexity condition (*p* < .001), as expected. Moreover, we found neither a significant main effect of the judge type (i.e., artificial intelligence, human; *F*(1, 602) = 0.15, *p* = .70) nor an interaction effect between the judge and case complexity type (*F*(2, 602) = 0.83, *p* = .44).

**Fig. 1:**
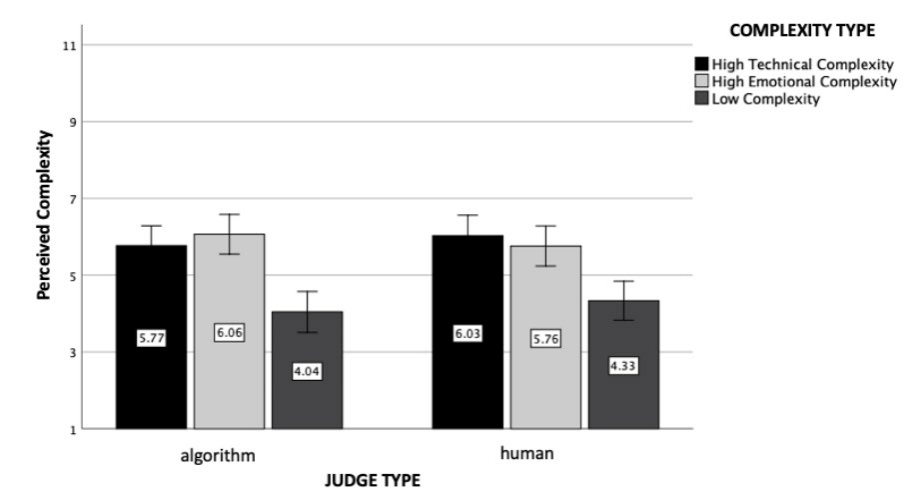
*Perceived Complexity*: The figure represents perceived complexity as a function of judge and case complexity type (experiment 1). Standard errors are represented in the figure by the error bars attached to each column

### Perceived Trust

**Fig. 2:**
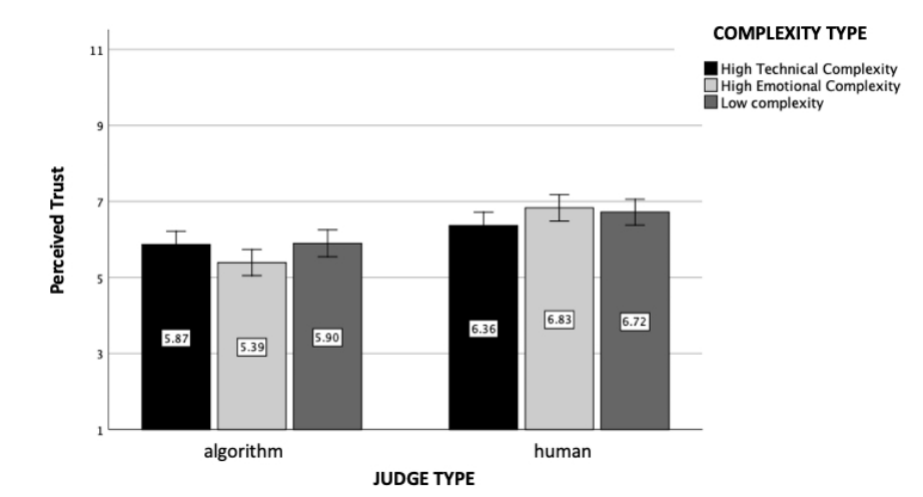
*Perceived Trust*: The figure represents perceived trust as a function of judge and case complexity type (experiment 1). Standard errors are represented in the figure by the error bars attached to each column

We found a significant main effect of the type of judge (*F*(1, 602) = 42.00, *p* < .001, η_p_^2^ = 0.07). Participants perceived the human judge to be more trustworthy (M = 6.64, SD = 1.49) than the algorithmic judge (M = 5.71, SD = 1.98). The main effect of case complexity was found non-significant (*F*(2, 602) = 0.84, *p* = .43). Importantly, the interaction effect between case complexity and judge type was significant (*F*(2, 602) = 3.83, *p* = .02, η_p_^2^ = 0.01, see Fig. 2). Even though participants generally trusted the algorithmic judge less than the human judge, individuals’ level of trust depended on the type of case complexity. Participants were found to trust the algorithm even less when the case included emotional complexities compared to the simple case (*p* = .04). This effect, however, was non-significant for the technically complex case (*p* = .91).

### Intentions

**Fig. 3:**
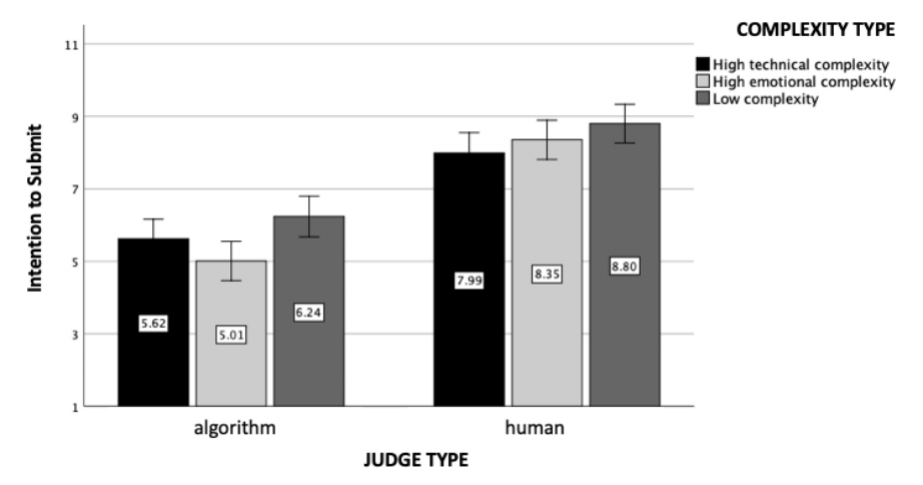
*Intention To Submit The Case*: The figure represents intentions to submit the case to the local court as a function of the judge and case complexity type (experiment 1). Standard errors are represented in the figure by the error bars attached to each column

A 2 (judge type) x 3 (case complexity type) ANOVA revealed a large main effect of judge type (*F*(1, 602) = 152.30, *p* < .001, η_p_^2^ = 0.20, see Fig. 3). Participants were more willing to submit their cases to the local court when the judge was human (M = 8.39, SD = 2.11) than when it was an algorithm (M = 5.61, SD = 3.32). The main effect of complexity type was also found to be significant (*F*(2, 602) = 5.47, *p* = .004, η_p_^2^ = 0.02): Participants were more willing to submit their cases when the case they read about was low in complexity (M = 7.58, SD = 3.01) than high in emotional complexity (M = 6.67, SD = 3.32; *p* = .002) or high in technical complexity (M = 6.77, SD = 2.90; *p* = .01). Although directionally similar to the results for perceived trust, the interaction effect between judge and case complexity type was non-significant (*F*(2, 602) = 1.78, *p* = .17).

### Perceived Speed

**Fig. 4:**
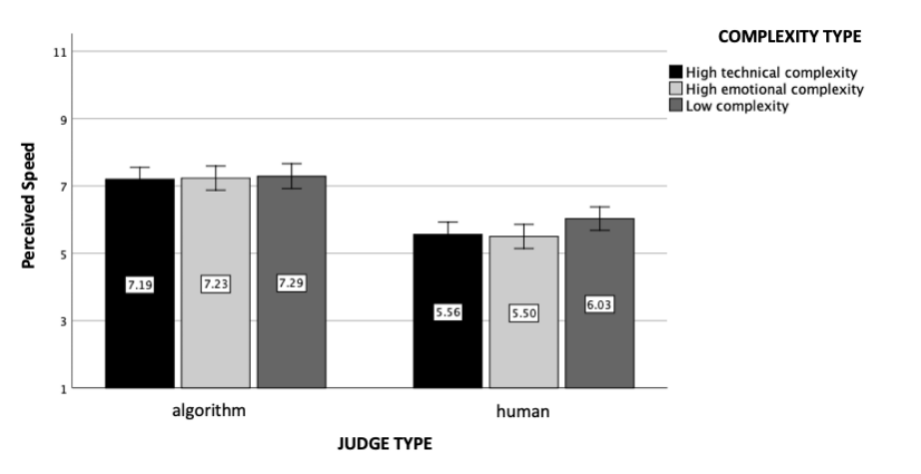
*Perceived Speed*: The figure represents perceived speed as a function of judge and case complexity type (experiment 1). Standard errors are represented in the figure by the error bars attached to each column

The analysis revealed a significant main effect of judge type (*F*(1, 602) = 110.29, *p* < .001, η_p_^2^ = 0.16, see Fig. 4): Participants perceived the human judge to be slower (M = 5.70, SD = 1.82) than the algorithmic judge (M = 7.24, SD = 1.80). Additionally, neither the main effect of the case complexity (*F*(2, 602) = 1.70, *p* = .18) nor the interaction effect was found to be statistically significant (*F*(2, 602) = 0.97, *p* = .38).

### Perceived Cost

**Fig. 5:**
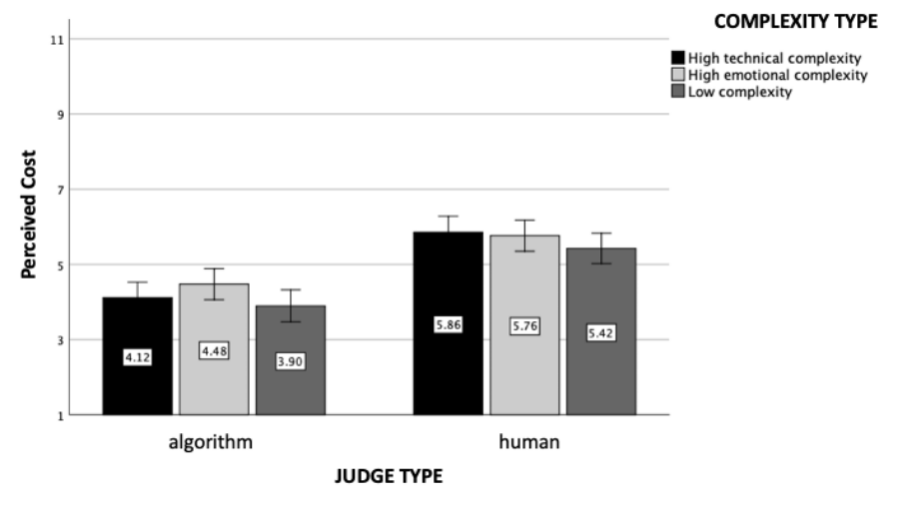
*Perceived Cost*: The figure represents perceived cost as a function of judge and case complexity type (experiment 1). Standard errors are represented in the figure by the error bars attached to each column

The main effect of judge type was significant (*F*(1, 602) = 80.17, *p* < .001, η_p_^2^ = 0.12, see Fig. 5). Participants perceived the algorithmic judge to be cheaper (M = 4.17, SD = 2.32) than the human judge (M = 5.68, SD = 1.84). Furthermore, the main effect of case complexity type was marginally significant (*F*(2, 602) = 2.60, *p* = .08, η_p_^2^ = 0.01): Cases with low complexities were perceived to be cheaper (M = 4.69, SD = 2.33) than the emotionally complex ones (M = 5.12, SD = 2.21, *p* = .03). Finally, the interaction effect between judge and case complexity type was revealed to be non-significant (*F*(2, 602) = 0.59, *p* = .56).

## Discussion of Experiment 1

Results of experiment 1 provide support for the notion that individuals care about the specific judge (human vs. algorithm) that will adjudicate their case. In the context of a divorce procedure, we find that individuals have lower intentions to go to their local courts when they are informed that an algorithm will adjudicate. This is a large effect (*d* = 1.0). With regard to trust, we find a similar pattern: human judges are trusted more than algorithms. Note that our comparisons are made relatively (algorithm versus human), and not in absolute terms.

Furthermore, the analysis on perceived trust indicates that also the type of case complexity matters as well. In particular, our results show that algorithmic judges are trusted even less when the complexity of the case derives from psychological factors (vs. low complexity vs. high technical complexity). Therefore, citizens might be relatively more open to algorithmic judges when they perceive high levels of technical complexity. Considering the perceived speed and cost, our findings validate the idea that individuals expect artificial intelligence to be faster and cheaper than humans. We find that this reluctance to go to court when the judge is not a human was not dependent on the type of case complexity (low vs. high emotional vs. high technical complexity). Apparently, the judge cue had such a strong impact on intentions that the information about the complexity of the case had no residual effect.

Overall, experiment 1 paints a rich picture of how court users think about the role of technology in the legal process and are likely to respond to the introduction of algorithms in the courtroom.

## Experiment 2

### Design and Participants

We recruited 1,214 American Mturkers (M_age_= 38.1, 52.9% F) in experiment 2. We used experiment 1’s design and randomly assigned participants to one of the six experimental conditions (judge type x case complexity type). Please see our Supplemental Materials for more details on the experimental stimuli, measures, and for details about randomization. Experiment 2 was pre-registered (please refer to http://aspredicted.org/blind.php?x=ap82nz for the preregistration plan).

### Materials and Procedure

Experiment 2 used experiment 1’s scenario: we again told participants to imagine that they and their partner agreed to separate. We then manipulated the complexity of the divorce case (low complexity vs. emotional vs. technical complexity) as well as the type of judge that would take their case (algorithmic versus human judge).

We also utilized the same measures used in experiment 1. The only exception was that we used two items to measure intentions in experiment 2 (i.e., “How likely would you be to submit your case that will be resolved by the artificial intelligence (vs. judge) to the local court?”, “In this situation, would you plan to submit your case that will be resolved by the artificial intelligence (vs. judge) to the local court?”; 1 = *not at all likely / no intention to submit* to 11 = *very likely / very strong intention to submit*; α = 0.91).

## Results of Experiment 2

### Manipulation check

**Fig. 6:**
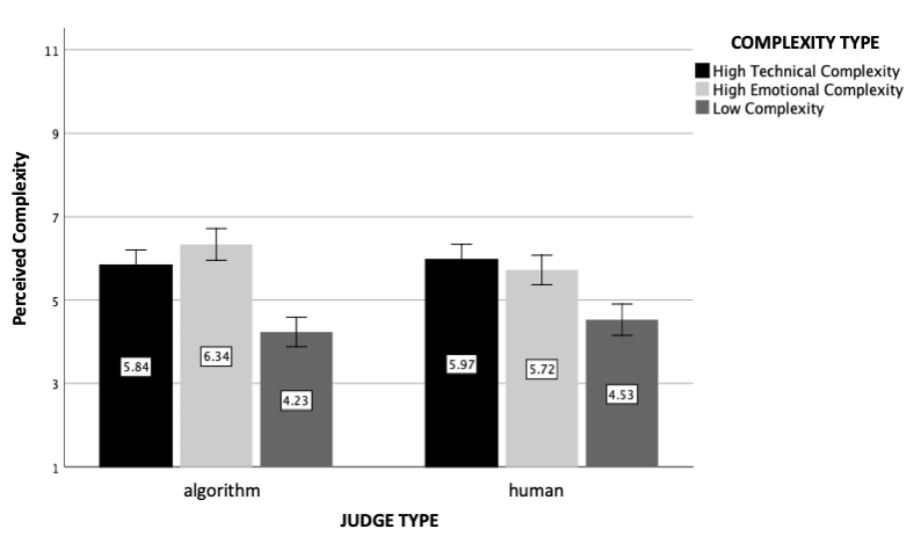
*Perceived Complexity*: The figure represents perceived complexity as a function of judge and case complexity type (experiment 2). Standard errors are represented in the figure by the error bars attached to each column

As expected, the main effect of case complexity was again statistically significant (*F*(2, 1208) = 50.39, *p* < .001, η_p_^2^ = 0.08), and the contrast between high emotional and high technical complexity case conditions was non-significant (*p* = .50), indicating that participants perceived the complexity of these cases the same regardless of its cause. Replicating experiment 1, the contrast analysis also revealed that both types of high complexity cases were perceived to be more complex than the low complexity one (*p* < .001). The main effect of type of the judge was found to be non-significant (*F*(1, 1208) = 0.16, *p* = .69). The interaction effect between the complexity and judge type, however, was significant this time (*F*(2, 1208) = 3.48, *p* = .03, η_p_^2^ = 0.006). This interaction effect indicates that perceived complexity of a simple divorce case was greater for a human judge compared to an algorithmic judge (*p* = .002, see Fig. 6), whereas the contrast between the two types of complex cases was statistically non-significant. Given that the materials were identical in the two studies and that the pattern of results in experiment 2 closely mimics those of experiment 1 (where we did not observe such an effect), this interaction on the manipulation check items is unlikely to explain the findings for the main dependent variables.

### Perceived Trust

**Fig. 7:**
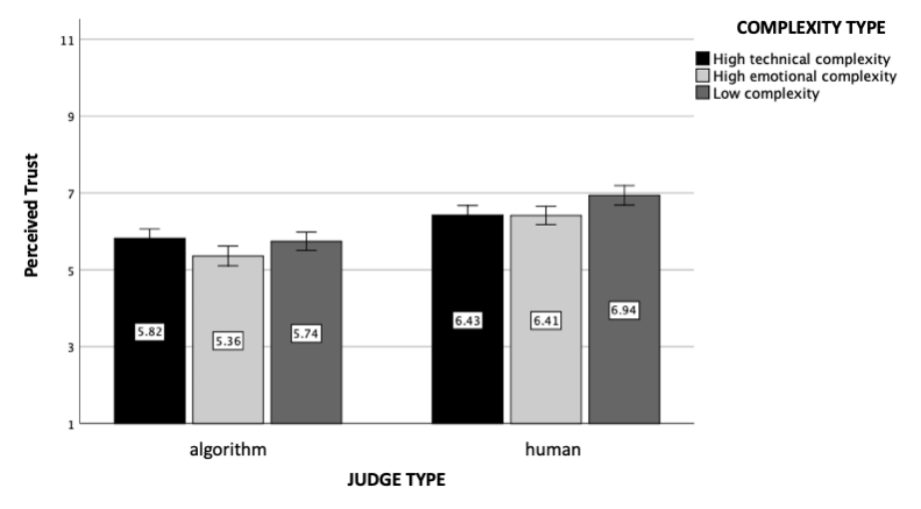
*Perceived Trust*: The figure represents perceived trust as a function of judge and case complexity type (experiment 2). Standard errors are represented in the figure by the error bars attached to each column

As pre-registered, we found a main effect of the judge type (*F*(1, 1208) = 89.51, *p* < .001, η_p_^2^ = 0.07): Participants perceived the human judge to be more trustworthy (M = 6.58, SD = 1.60) than the algorithmic judge (M = 5.65, SD = 1.91). Furthermore, the main effect of case complexity was also significant in this study (*F*(2, 1208) = 6.72; *p* = .001, η_p_^2^ = 0.01): Participants who read about the low complexity case perceived the judge as more trustworthy (M = 6.3, SD = 1.86) than participants who read about the emotionally (M = 5.93, SD = 1.85; *p* < .001) or technically complex cases (M = 6.12, SD = 1.74; *p* = .08). Importantly, the interaction effect between complexity and judge type was again significant (*F*(2, 1208) = 3.12, *p* = .04, η_p_^2^ = 0.005, see Fig. 7). Similar to the results of experiment 1, participants trusted the algorithm even less when the case included emotional complexities compared to cases that were low in complexity (*p* = .03) or technically complex (*p* = .01). Interestingly, participants trusted the human judge more when the case was uncomplicated compared to cases that were high in emotional (*p* = .003) or technical complexity (*p* = .004).

### Intentions

**Fig. 8:**
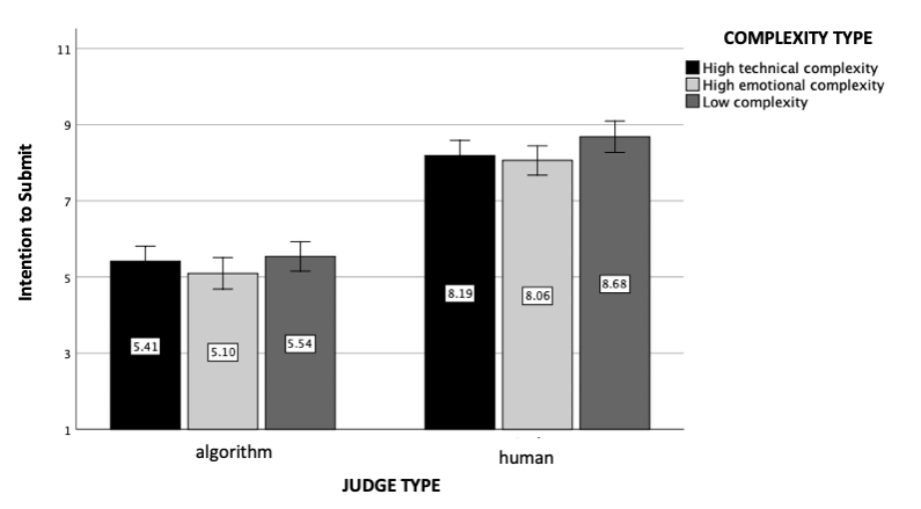
*Intention To Submit The Case*: The figure represents intentions to submit the legal case to the local court as a function of judge and case complexity type (experiment 2). Standard errors are represented in the figure by the error bars attached to each column

As pre-registered, a 2 (judge type) x 3 (case complexity type) ANOVA revealed an even stronger main effect of judge type (*F*(1, 1208) = 331.40, *p* < .001, η_p_^2^ = 0.22, see Fig. 8). Replicating experiment 1’s pattern, participants were more willing to submit their cases when the judge was human (M = 8.3, SD = 2.34) than an algorithm (M = 5.36, SD = 3.25). The main effect of case complexity was again statistically significant (*F*(2, 1208) = 3.61, p = .03, η_p_^2^ = 0.006): Participants were more willing to submit their cases when the case they read about was low in complexity (M = 7.01, SD = 3.2) than high in emotional complexity (M = 6.69, SD = 3.32; *p* = .008). This contrast was only directional when comparing the cases with low and high technical complexity (M = 6.8, SD = 3.04; *p* = .12). Finally, the interaction between judge type and case complexity was non-significant (*F*(2, 1208) = 0.42, *p* = .66), indicating that interaction observed for trust did not spill over to intentions.

### Perceived Speed

**Fig. 9:**
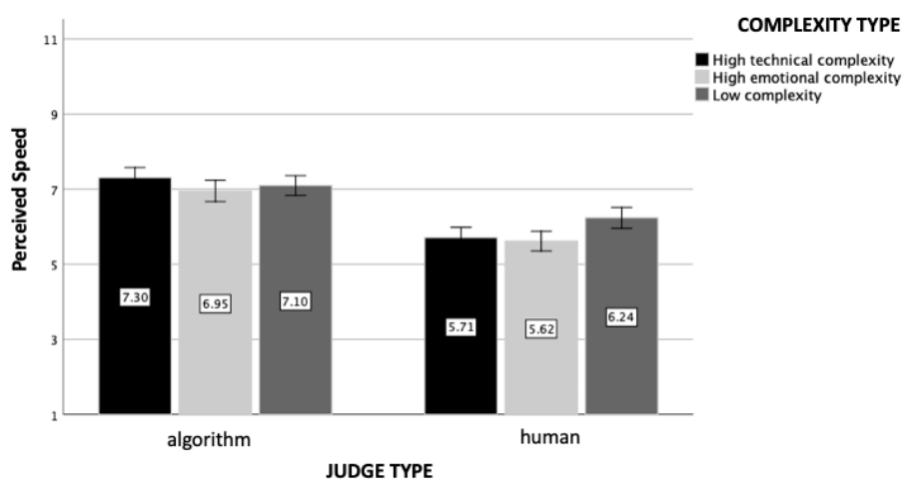
*Perceived Speed*: The figure represents perceived speed as a function of judge and case complexity type (experiment 2). Standard errors are represented in the figure by the error bars attached to each column

Replicating experiment 1’s results, a 2 (judge type) x 3 (case complexity type) ANOVA revealed a significant main effect of judge type (*F*(1, 1208) = 129.48, *p* < .001, η_p_^2^ = 0.10, see Fig. 9). Participants again perceived the human judge to be slower (M = 5.84, SD = 1.93) than the algorithmic judge (M = 7.12, SD = 1.95). The main effect of case complexity was also significant (*F*(2, 1208) = 3.99, *p* = .02, η_p_^2^ = 0.007). In particular, cases that were low in complexity were considered to be processed faster (M = 6.69, SD = 1.95) than the ones that were emotionally complex (M = 6.23, SD = 2.05; *p* = .005). Finally, the interaction effect was significant (*F*(2, 1208) = 3.77, *p* = .02, η_p_^2^ = 0.006). Interpreting this interaction effect, human judge was perceived to be faster when the legal case was uncomplicated compared to emotionally (*p* = .001) or technically complex legal cases (*p* = .007), with no such difference in the case of the algorithmic judge (*p* > .27).

### Perceived Cost

**Fig. 10:**
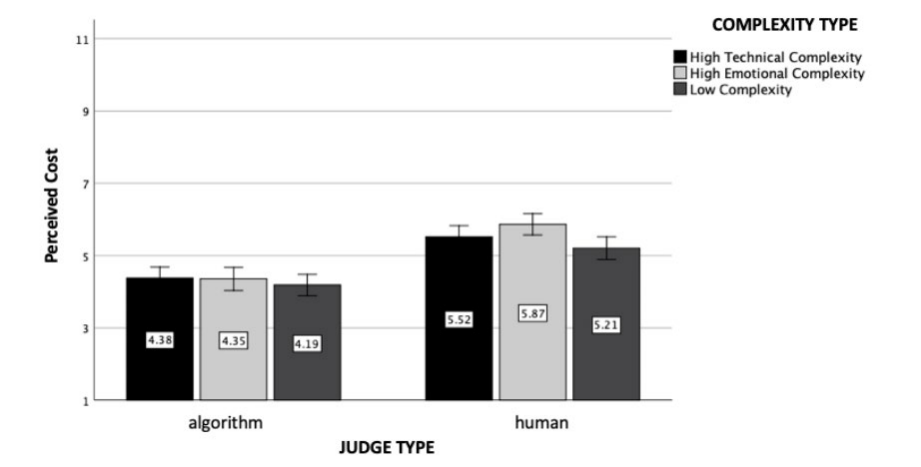
*Perceived Cost*: The figure represents perceived cost of the legal case as a function of judge and case complexity type (experiment 2). Standard errors are represented in the figure by the error bars attached to each column

Replicating experiment 1’s results, the main effect of the judge type was statistically significant (*F*(1, 1208) = 96.33, *p* < .001, η_p_^2^ = 0.07, see Fig. 10). Human judge was again perceived to be more expensive (M = 5.54, SD = 2.03) than the algorithmic judge (M = 4.30, SD = 2.32). Furthermore, the main effect of the case complexity was also significant (*F*(2, 1208) = 3.74, *p* = .02, η_p_^2^ = 0.006): Participants who read about the uncomplicated case rated the perceived cost to be significantly lower (M = 4.66, SD = 2.32) than the ones who read about emotionally (M = 5.17, SD = 2.21; *p* = .007) or technically complex cases (M = 4.95, SD = 2.23; *p* = .097). The interaction effect between judge and case complexity type was again non-significant (*F*(2, 1208) = 1.39, *p* = .25).

11. **Discussion of Experiment 2**..

The results of experiment 2 replicate the key main effects of judge type from experiment 1: Respondents reported less trust and lower intentions to submit a legal case to the local court when the judge was an algorithm than when it was a human. These main effects of judge type were again large in magnitude (intentions: *d* = 1.04; perceived trust: *d* = 0.53), corroborating generally negative views of respondents towards algorithmic judges. Moreover, we replicated the results for perceived speed and cost: The algorithmic judge was perceived to be faster and cheaper than the human judge. Finally, replicating experiment 1’s findings, we observed an interaction between judge and case complexity type on perceived trust.

12. **General Discussion**.

Every day, more and more computational and predictive technologies are being used within social institutions, including the justice system. There are many ongoing discussions about how to integrate AI in judicial decision-making and justice is one of the most frequently mentioned domains in which algorithms have a high potential to change the current practices (Araujo et al. 2018). We argue that it is important to understand how individuals perceive algorithmic judges when discussing the future application of AI in deciding court cases.

The current work studies individuals’ trust towards algorithmic and human judges and explores their intentions to submit their cases to a local court. In two empirical studies with a combined sample of over 1,800 adult US residents, we provide strong support for the notion that individuals care about the specific judge (human vs. algorithm) that will adjudicate their case. Specifically, we demonstrate that even though potential court users acknowledge that algorithms might lead to quicker and cheaper processes, perceived trust, and willingness to submit a case to court is negatively influenced by the use of algorithmic judge. Moreover, although human judges are in general trusted much more than algorithmic judges, both technical and emotional complexities reduce trust in human judges, whereas only emotional complexities reduce trust in algorithmic judges.

To provide robustness of our findings, we combined the data from all studies we ran in a single data file (three studies in total) and meta-analysed the findings (N = 3,039, M_age_ = 37.8, 53.00% F). We again found for trust a significant main effect of judge type (*F*(1, 3021) = 238.94, *p* < .001, η_p_^2^ = 0.07, *d* = 0.6) and type of case complexity (*F*(2, 3021) = 7.85, *p* < .001, η_p_^2^ = 0.005). Importantly, in line with the results of experiments 1 and 2, we found a significant interaction effect between judge and case complexity type on perceived trust (*F*(2, 3021) = 4.34, *p* = .01, η_p_^2^ = 0.003). Details of this internal meta-analysis can be found in the Supplemental Materials.

Our work provides novel insights on the impact of algorithms on individuals’ attitudes and decision-making. First, we document algorithm aversion in an important domain: Judicial decision-making. In many situations people need to go to court to protect their rights. The idea of facing an algorithmic judge may increase their frustration and influence their predisposition to use courts. Therefore, access to justice may suffer. Accordingly, despite the positive aspects of algorithms (i.e., speed and cost), policy-makers should expect pushback from citizens against courts’ adoption of algorithms in adjudication.

Our paper also adds to the growing literature on algorithmic decision-making (Helberger et al. 2020; Yeomans et al. 2019), we document its effect in a practical context, perceived trust of algorithmic and human judges. Additionally, existing research on algorithm aversion predominantly studies how individuals choose between using algorithms and humans (Dietvorst et al. 2018; Dietvorst et al. 2015). We contribute to this line of research by investigating how individuals perceive algorithms and humans when they are on the receiving side of the decisions that would be made by such decision-makers. Finally, our paper adds to the existing work on algorithms as we investigate the impact of legal case complexity (emotional vs. technical complexity). In particular, results of our internal meta-analysis highlight that trust in algorithmic judges especially drops when a legal case involves emotional complexity (vs. technical vs. low complexity).

13. **Limitations and Future Directions**.

Our studies have several limitations that deserve attention. First, all our respondents were US residents. Therefore, we would advise policy-makers not to generalize our results to respondents residing in other countries as it is possible that differences across countries may influence the general trust in judges. For instance, in countries with low court trust and low esteem of justice institutions, algorithmic judges may be trusted more than in countries in which courts and the justice administration have a better reputation. In addition to trust in the judicial system, court users’ trust in courts is influenced also by other factors, such as legal culture, the case at hand, the presence of a lawyer, or previous experiences. Future research is needed to conduct the same research in other jurisdictions and to use court or justice trust indicators when comparing data between jurisdictions. Second, trust in algorithmic decisions might also be influenced by repeated interaction with an algorithmic judge. For instance, experienced court players may have different attitudes towards algorithmic judges as they practice. In addition, we concur with Rule and Friedberg that trust in an algorithm should be considered in the broader context of where, how, and when the algorithm is used to resolve conflicts (Rule and Friedberg 2005). Trust is a contextual construction. We recommend more research on the effect of repeated exposure to algorithmic judges.

Third, even though there are many differences between humans and algorithms, current work aims to study lay people’s general perceptions of algorithms in judicial decision-making. Therefore, we prioritized achieving high internal validity and minimized differences between conditions by only manipulating the type of judge. Future research should investigate differences between algorithmic and human judges systematically. Research should focus also on hybrid situations where AI and humans work together, for instance an AI system supports the judge to draft a decision, or an AI system and a judge write a decision together. The level of AI integration and its relation to human judges may take many shapes and may affect people differently. Additionally, our paper covers several different perceptions such as trust, speed, and cost. However, we do not investigate how and when these variables impact individuals’ decisions to submit their legal cases to the court. More research is needed to further understand the dynamics between perceptions of algorithms and their impact on individuals’ attitudes and behaviours.

Further research might also delve deeper into the potential differences between legal fields. Depending on the field of law and the type of case, there might be divergence in the legal knowledge and in the approach potential court users take. These differences can be explained by the fact that parties are assisted by legal professionals like attorneys, who exercise considerable power over their clients and control their litigation strategies (Themeli 2018). Moreover, differences in the nature of the parties (e.g., business vs. private individuals) might have an influence on the willingness to submit a case to an algorithmic judge.

Our research is comparable to that of Sela (2018). Both our studies indicate less appreciation for automated decision-making. However, the studies differ in the dispute resolution mechanism under investigation – court for us, ODR for Sela (2018); and the timing of the interview– ex ante for us, ex post for Sela (2018). Additionally, we investigate the role of different types of case complexities to provide policy-makers with insights about what to expect when they adopt algorithmic judges.

In addition, our research may be comparable to Helberger et al. (2020). Both our studies investigate human perception of algorithm (*automated* for Helberger et al.) decision-makers but reach different conclusions. This may be due to the following difference between both studies: Helberger et al. (2020) survey is broad and without reference to any sector, whereas our experiment focuses on court litigation; Helberger et la. inquire on the perception of fairness (as used in legal literature), whereas for us fairness is one of the elements that constitute trust; Helberger et al. base their study on a survey, ours is an experiment which uses complexity moderators in addition to manipulating human vs. algorithm; Helberger et al. use a Dutch sample, whereas our sample is based in the US. Nevertheless, both our studies agree that the mechanism with which humans perceive algorithmic decision-makers is complex and sensitive to circumstances. Both our studies agree that more studies are needed in this direction.

Finally, we are also aware that the underlying values and concepts in this paper are very much legally imprinted. Our use of the categories simple and complex is closely related to what is accepted as such in the legal world (Themeli and Philipsen 2021). A civil litigation is legally simple when parties compromise on the outcome and the judge only has to sign at the bottom, after a marginal assessment of compatibility with minimum standards of law. In the psychological and technological frame of concepts and values, the categories simple and complex might refer to something totally different. Consequently, legally simple is not equal to easy to automate. To find out how those differences play out, a conversation is needed on the intricate conventions between the disciplines (de Vey Mestdagh 2020). Then it may turn out that the legally simple cases comprise a much larger variation in complexity than we envisage and that complex in the legal world does not correspond with complex in the technical world. We observe that behind a simple court case often a host of human complexities are hidden. We tried to mitigate the effects of our respective imprints, at least in part, by composing a multidisciplinary team for this first investigation. To bring our results further to concrete policy guidelines requires the inclusion of other experts into the conversation.

## Electronic Supplementary Material

Below is the link to the electronic supplementary material.

Supplementary Material 1
