# Supplementary material for: Perceptions of Justice By Algorithms
Source: Artif Intell Law (Dordr). 2022 Apr 5;31(2):269–92. doi: 10.1007/s10506-022-09312-z (PMC10102053; doi:10.1007/s10506-022-09312-z)
Supplement: Supplementary file 1 — Supplementary Material 1 [file 10506_2022_9312_MOESM1_ESM.docx]

# Supplemental Materials

## Supplemental Material For Experiment 1

### Scenario

This survey is about decision making in courts [This survey is about decision making by algorithms and artificial intelligence in the courts]. Suppose you have been married for some years. Lately, you and your partner feel that the love for each other has cooled down to almost zero. You agree to separate and file for divorce.

**High technical complexity:** You and your partner jointly own a house and some savings. You pay for 65% of the costs of daily living because your salary is much higher than that of your partner. On the other hand, 20% of the house was paid out of an inheritance that your partner had obtained after your partner’s grandma passed away. The rest was paid out of a mortgage. It is very likely that for your partner, it will be hard to uphold the same standard of living after the divorce. For the divorce, you go to your local court.

**High emotional complexity:** You and your partner jointly own a house and some savings. The costs of your daily life are covered on an equal basis out of your salaries, where you both have full-time jobs. Your partner’s mental health has suffered from the negative development in the marriage. Your partner has experienced many sleepless nights worrying about the future and suffered a nervous breakdown. As a result, your partner cannot go to work. You feel somewhat sad about the whole breakup and the impact it has on your partner, but at the same time look forward to a new life on your own. For the divorce, you go to your local court.

**Low complexity**: You and your partner jointly own a house and some savings. The costs of your daily life are covered on an equal basis out of your salaries, where you both have full-time jobs. For the divorce, you go to your local court.

Cases like yours are resolved by an experienced judge from the local court [At your local court, a new system has been in place for some time now, where cases are resolved by artificial intelligence and algorithms. Cases like yours are resolved by this new system, which is fully automated and uses the legislation and the relevant case law of your jurisdiction to resolve disputes.]

### Measures

- **Perceived trust (four-item):** Thinking about this divorce case and your future court experience, to what extent do you think that the judge [artificial intelligence] will be (1 = unfair / not trustworthy / unpredictable / biased to 9 = fair / trustworthy / predictable / unbiased)
- **Perceived speed:** Thinking about this divorce case and your future court experience, to what extent do you think that the judge [artificial intelligence] will be (1 = slow to 9 = fast)
- **Perceived cost:** Thinking about this divorce case and your future court experience, to what extent do you think that the judge [artificial intelligence] will be (1 = expensive to 9 = cheap)
- **Intentions to submit the case**: How likely would you be to submit your case that will be resolved by the judge [artificial intelligence] to the local court? (1 = not at all likely to 11 = very likely)
- **Manipulation check (two-item)**: When you think about the case that you read, how complicated do you think this divorce case is? and How complicated do you think this divorce case is for judge [artificial intelligence] to resolve? (1 = not at all complicated to 11 = very complicated)
- **Experience in courts:** How experienced are you in courts? (1 = completely unexperienced to 7 = completely experienced)
- **ICT:** To what extent are you an experienced user of ICT (information and communications technologies)? (1 = completely unexperienced to 7 = completely experienced)
- **Age**
- **Gender** (0 = male, 1 = female, 2 = other, 3 = prefer not to answer)
- **Marital status** (1 = married, 2 = widowed, 3 = divorced, 4 = separated, 5 = never married)
- **Education**: What is the highest degree that you have completed? (1 = less than high school to 7 = doctorate)
- **Income:** Approximately how much income do you personally make per year? (1 = less than $20,000 to 10 = over $250,000, 11 = prefer not to share)
- **Open-ended question:** Any comments?

In experiment 1, we found no systematic differences across conditions in terms of participants’ age, gender, income, marital status, the extent that they were experienced in courts, and the extent that they use information consumer technologies (ICT). The only exception to this was that participants’ experience in using ICT (*F*(1, 602) = 8.32, *p* = .004) differed depending on the judge condition that participants were assigned to. Importantly, controlling for this variable did not change the results for neither of our key variables (i.e., perceived trust, intentions to submit the case, perceived speed, perceived cost).

Additionally, experiment 1 included an item to measure how negative participants perceive the relationship to be (“Based on the scenario that you read, how negative or compromised do you think that the relationship between you and your partner is?”; 1 = *extremely negative* to 7 = *extremely* *positive*). A 2 (judge type) x 3 (case complexity type) ANOVA revealed that the perceived negativity between parties was not perceived to be different depending on the judge (AI vs. human; *F*(1, 602) = .02, *p* = .90). Additionally, we found a significant main effect of type of case complexity (*F*(2, 602) = 9.52, *p* < .001): The relationship was perceived to be more negative when the case was high in emotional complexity (M = 2.88, SD = 1.34) compared to the cases that were uncomplicated (M = 3.32, SD = 1.41; *p* = .002) or high in technical complexity (M = 3.45, SD = 1.42; *p* < .001). The interaction effect between the judge and case complexity type was not statistically significant (*F*(2, 602) = .67, *p* = .51).

## Supplemental Material For Experiment 2

### Scenario

This survey is about decision making in courts [This survey is about decision making by algorithms and artificial intelligence in the courts]. Suppose you have been married for some years. Lately, you and your partner feel that the love for each other has cooled down to almost zero. You agree to separate and file for divorce.

**High technical complexity:** You and your partner jointly own a house and some savings. You pay for 65% of the costs of daily living because your salary is much higher than that of your partner. On the other hand, 20% of the house was paid out of an inheritance that your partner had obtained after your partner’s grandma passed away. The rest was paid out of a mortgage. It is very likely that for your partner, it will be hard to uphold the same standard of living after the divorce. For the divorce, you go to your local court.

**High emotional complexity:** You and your partner jointly own a house and some savings. The costs of your daily life are covered on an equal basis out of your salaries, where you both have full-time jobs. Your partner’s mental health has suffered from the negative development in the marriage. Your partner has experienced many sleepless nights worrying about the future and suffered a nervous breakdown. As a result, your partner cannot go to work. You feel somewhat sad about the whole breakup and the impact it has on your partner, but at the same time look forward to a new life on your own. For the divorce, you go to your local court.

**Low complexity**: You and your partner jointly own a house and some savings. The costs of your daily life are covered on an equal basis out of your salaries, where you both have full-time jobs. For the divorce, you go to your local court.

Cases like yours are resolved by an experienced judge from the local court [At your local court, a new system has been in place for some time now, where cases are resolved by artificial intelligence and algorithms. Cases like yours are resolved by this new system, which is fully automated and uses the legislation and the relevant case law of your jurisdiction to resolve disputes.]

### Measures

- **Perceived trust (four-item):** Thinking about this divorce case and your future court experience, to what extent do you think that the judge [artificial intelligence] will be (1 = unfair / not trustworthy / unpredictable / biased to 9 = fair / trustworthy / predictable / unbiased)
- **Perceived speed:** Thinking about this divorce case and your future court experience, to what extent do you think that the judge [artificial intelligence] will be (1 = slow to 9 = fast)
- **Perceived cost:** Thinking about this divorce case and your future court experience, to what extent do you think that the judge [artificial intelligence] will be (1 = expensive to 9 = cheap)
- **Intentions to submit the case (two-item)**: “How likely would you be to submit your case that will be resolved by the judge [artificial intelligence] to the local court?” (1 = not at all likely to 11 = very likely) and “In this situation, would you plan to submit your case that will be resolved by the judge [artificial intelligence] to the local court?” (1= no intention to submit to 11= very strong intention to submit)
- **Manipulation check (two-item)**: “When you think about the case that you read, how complicated do you think this divorce case is?” and “How complicated do you think this divorce case is for judge [artificial intelligence] to resolve?” (1 = not at all complicated to 11 = very complicated)
- **Experience in courts:** How experienced are you in courts? (1 = completely unexperienced to 7 = completely experienced)
- **ICT:** To what extent are you an experienced user of ICT (information and communications technologies)? (1 = completely unexperienced to 7 = completely experienced)
- **Age**
- **Gender** (0 = male, 1 = female, 2 = other, 3 = prefer not to answer)
- **Marital status** (1 = married, 2 = widowed, 3 = divorced, 4 = separated, 5 = never married)
- **Education**: What is the highest degree that you have completed? (1 = less than high school to 7 = doctorate)
- **Income:** Approximately how much income do you personally make per year? (1 = less than $20,000 to 10 = over $250,000, 11 = prefer not to share)
- **Open-ended question:** Any comments?

In experiment 2, we again observed no systematic differences across conditions in terms of participants’ age, gender, income, marital status, the extent that they were experienced in courts, and the extent that they use information consumer technologies (ICT). There were two exceptions. First, participants’ experience in using ICT (*F*(1, 1208) = 9.97, *p* = .002) was again found to be different depending on the judge condition. Second, participants’ level of education marginally differed depending on the case complexity condition that they were assigned to (*F*(1, 1208) = 2.80, *p* = .06). Importantly, controlling for these two items did not change the results of the key variables we reported (i.e., perceived trust, intentions to submit the case, perceived speed, perceived cost).

In experiment 2, we measured how negative participants perceive the relationship to be (“Based on the scenario that you read, how negative or compromised do you think that the relationship between you and your partner is?”; 1 = *extremely negative* to 7 = *extremely positive).* We found that the main effect of the judge type was non-significant (AI vs. human; *F*(1, 1208) = .85, *p* = .36). Moreover, we observed a significant main effect of type of case complexity (*F*(2, 1208) = 20.70, *p* < .001): participants perceived the relationship to be more negative when the legal case included emotional complexities (M = 2.92, SD = 1.34) than technical complexities (M = 3.29, SD = 1.38; *p* < .001) or no complexities (M = 3.54, SD = 1.45; *p* < .001). The contrast between high technical complexity and low complexity conditions was also shown to be significant (*p* = .007). Note that the interaction effect between the type of judge and complexity type was also statistically significant (*F*(2, 1208) = 4.286, *p* = .014).

## Supplemental Material For The Internal Meta Analysis

In addition to the two studies reported in the paper, we conducted another study (N = 1,217 US residents, M_age_ = 37.4, 54.2% F) on Amazon Mturk for this research project. Participants were asked to imagine that they bought a second-hand car from Tempra Car Dealers. The car suddenly stopped working on the next day, but the car dealer was told to deny responsibility. Similar to other studies, participants were again randomly assigned to one condition of a 2 (judge type: algorithm vs. human) x 3 (case complexity type: low complexity vs. high emotional complexity vs. high technical complexity) and used the same items we measured in experiments 1 and 2. The reason that this study was not included in the paper is that our manipulation for case complexity failed. Specifically, participants did not perceive the emotionally (M = 5.87, SD = 2.74) and technically (M = 5.54, SD = 2.62) complex cases equally complex as intended (*p* < .05).

Even though this study had problems as our manipulation check failed, to test the robustness of this effect and to show that we do not have a file drawer, we combined the data from all three studies in a single data file and submitted the data to an analysis similar to those reported above to test the robustness of the effects above regarding the trust perceptions of participants after reading about different types of judges (i.e., artificial intelligence, human) and different types of case complexity (i.e., low complexity, high technical complexity, high emotional complexity). Note that we have no file drawer, these are all the data collected for this paper.

In this internal meta-analysis, we tested participants’ reactions to different types of judges (algorithmic versus human) in three different studies, with 3,039 participants in total (M_age_ = 37.8, 53.00 % F). Aggregating these three studies, we again found for trust a significant main effect of judge type (*F*(1, 3021) = 238.94, *p* < .001, η_p_^2^ = .07, *d* = .6) and type of case complexity (*F*(2, 3021) = 7.85, *p* < .001, η_p_^2^ = .005). Importantly, we found a significant interaction effect between judge and case complexity type on perceived trust (*F*(2, 3021) = 4.34, *p* = .01, η_p_^2^ = .003). This interaction effect was in line with what we described in experiments 1 and 2. For instance, zooming into the algorithm conditions, we again found that participants trusted the algorithm less when the case included emotional complexities compared to both simple cases (*p* = .001) and cases that are complex due to technicalities (*p* = .004). Interestingly, we did not observe a difference between simple and technically complex cases (*p* = .64), suggesting that technical complexity hurt algorithmic judges relatively less. Additionally, we found that participants trusted the human judge more when the case was uncomplicated compared to both technically (*p* = .001) and emotionally complex cases (*p* = .03). From these analyses we conclude that, although human judges are in general trusted much more than algorithmic judges, both technical and emotional complexities reduce trust in human judges, whereas only emotional complexities reduce trust in algorithmic judges.

Importantly, the three-way interaction with study as an additional factor is non-significant (*F*(4, 3021) = 1.71, *p* = .15), indicating that the magnitude of the interaction effect does not vary significantly across studies. This non-significance of the three-way interaction is especially noteworthy given the lar
